# Impacts of gain versus loss frame messages about beverages on boy students, an application of extended parallel process model

**DOI:** 10.1186/s41043-022-00301-1

**Published:** 2022-05-19

**Authors:** Fateme Zareharofteh, Masoud Karimi

**Affiliations:** 1 Shahid Sadoughi University of Medical Sciences, Yazd, Iran; 2 Department of Health Promotion, School of Public Health, Shiraz University of Medical Sciences, Al-Zahra Street, Shiraz, 14336-71348 Iran

**Keywords:** Sugar-sweetened beverages, Extended parallel process model, Gain frame, Loss frame, Intention

## Abstract

**Background:**

Unhealthy diet including consumption of high amounts of sugar-sweetened beverages is a key modifiable risk factor for obesity and NCDs which begin in childhood and adolescence. The study aimed to compare the effect of gain frame vs. loss frame messages on SSBs consumption intention and behavior of high school boy students.

**Methods:**

In this quasi-experimental study, 270 students from three boy’s high schools were selected through a multistage random sampling. Data collection was done through a 15 items self-reported questionnaire before and two months after the intervention. Each of the two intervention groups received one of the two types of gain frame or loss frame designed pamphlets inspired with extended parallel process model. The control group received no pamphlet.

**Results:**

In control, GFM and LFM groups 91, 86 and 89 students participated in the study, respectively. After the intervention, significant differences were observed in perceived efficacy and threat of the GFM group and perceived efficacy, threat and intention in the LFM group compared with before the intervention. The GFM group had higher perceived self-efficacy than the control group and lower perceived severity than the LFM group. Intention to consume SSBs reduced significantly in LFM group, compared with the control group.

**Conclusions:**

A combination of LFM and GFM messages could more effectively lead to nutritional behavior change regarding the consumption of SSBs. Results help to design messages for educational programs and nutritional campaigns.

## Introduction

Overweight and obesity during childhood and adolescence are associated with increased risk of non-communicable diseases (NCDs) in adulthood [1]. The global obesity epidemic is worsening in most parts of the world; the prevalence of obesity has doubled since 1980 [2]. It was estimated that 12.9% and 13.4% of adolescent boys and girls in developing countries were overweight and obese, respectively, in 2013 [3]. In a multi-centric, cross-sectional study on 12–18-year-old adolescents in 30 provinces of Iran in 2015, the prevalence rates of abdominal, generalized, and combined obesity were reported as 12.18%, 1.81%, and 9.24%, respectively [4].

Unhealthy diet is a key modifiable risk factor for obesity and NCDs, which begins in childhood and adolescence and builds up throughout the life [1, 5–7]. Sugar-sweetened beverages (SSBs, carbonated or noncarbonated beverages that contain high amounts of sugar and are flavored with natural or artificial additives, including regular soda, fruit drinks, and sports and energy drinks) are a leading source of added sugar to the diet among adults and children. Evidence has suggested that high consumption of SSBs was associated with excess energy intake and was strongly linked to obesity [8–10].

While the Dietary Guidelines for Americans have recommend limiting the intake of daily added sugars to less than 10% of total daily calories [11], the consumption of SSBs has recently increased in developing countries, such as Iran [9]*.* In one of the few studies performed in Iran, on average, 20.8% of the total daily energy was supplied by SSBs (98 and 70 ml/day in boys and girls, respectively) [12]. Furthermore, 37% of television advertisements between 14:00 and 21:00, i.e., the time most children tend to watch television, have been involved with soft drinks in the Middle East [13].

**Fig. 1:**
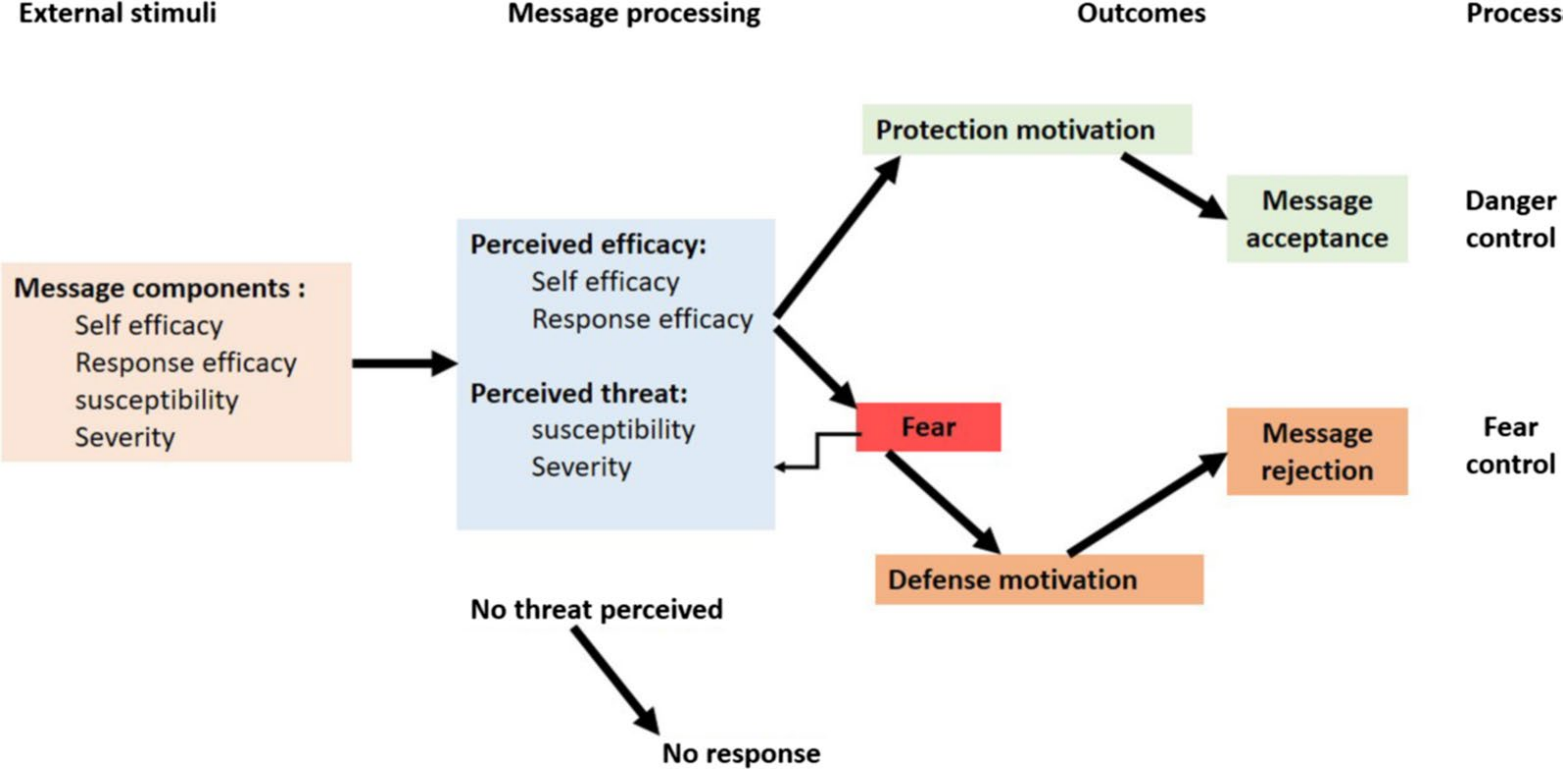
Extended parallel process model, Kim witte 1994

Since unhealthy diet including excessive consumption of SSBs is a modifiable health risk behavior [1]*,* trying to change the behavior of consumers to drink healthier beverages seems essential. To create effective health behavior, change interventions, evidence-based behavior change theories are important. Good theories give us the ability to predict and understand, at least in part, how and why behavior changes and allow for better intervention designs. Evidence has suggested that theory-based health education interventions can lead to more powerful effects compared to no theory driven Programs [14–16]_._ One of the useful models in this regard is the Extended Parallel Process Model (EPPM), which emphasizes the interaction between individuals’ emotions (perceived threat) and rationale (perceived efficacy) in decision-making for adopting health behaviors [17]. Based on the EPPM (Fig. 1), people confronted with health messages, such as pamphlets, Public Service Announcements (PSA), and billboards, make a decisional balance based on their perceived threat (susceptibility to health danger and its severity) and perceived efficacy (self-efficacy and response efficacy), leading to three possible responses: no response (low perceived susceptibility/severity appraisal), danger control response (high perceived susceptibility/severity and efficacy appraisal), and fear control response (high perceived susceptibility/severity but low efficacy appraisal). The danger control response is a cognitive process in which individuals intend to follow or do message recommendations. On the other hand, the fear control response is an emotional process in which people engage in defensive mechanisms, such as avoidance, denial, and reactance, aimed at reducing fear rather than lessening the threat [18–21].

One of the important factors leading audiences to accept or reject recommendations in health communication campaigns is message framing [17]. Some evidences have suggested that loss-framed threatening messages (focused on the costs or adverse effects of doing risky behaviors or not adopting healthy behaviors**)** that are widely used in health communications often resulted in fear control responses [22]. However, the results of the studies on different behaviors were inconsistent. For example, while gain-framed messages (focused on the positive outcomes of advised healthy behaviors) that emphasized the benefits of adhering to a health message recommendation tended to be more persuasive for engaging in behaviors, such as cancer screening and using condoms, sunscreen, and dental floss in some studies, loss-framed messages focused on the costs or consequences of high-risk behaviors or not adhering to a health behavior were more effective in persuading people to engage in behaviors, such as HIV testing and mammography, in other studies[23, 24]. Some researchers believed that the effects of gain- or loss-framed messages depended on the nature of behaviors. They argued that loss-framed messages affected high-risk behaviors, while gain-framed ones affected less risky behaviors, such as eating behaviors [25]. However, various studies have shown inconsistent results on the effect of message type on nutritional behaviors [26–29].

The consumption of SSBs has exceeded the recommendations among adolescents, especially boys, in Iran [12]. Besides, a review of the literature revealed that no previous research has investigated the effect of the EPPM-based interventions on SSBs consumption. Therefore, the present study aims to investigate the effects of two different types of messages (gain-framed vs. loss-framed) inspired by the EPPM on adolescent male students’ attitudes, intentions to consume SSBs, and consumption of SSBs.

## Materials and methods

**Fig. 2:**
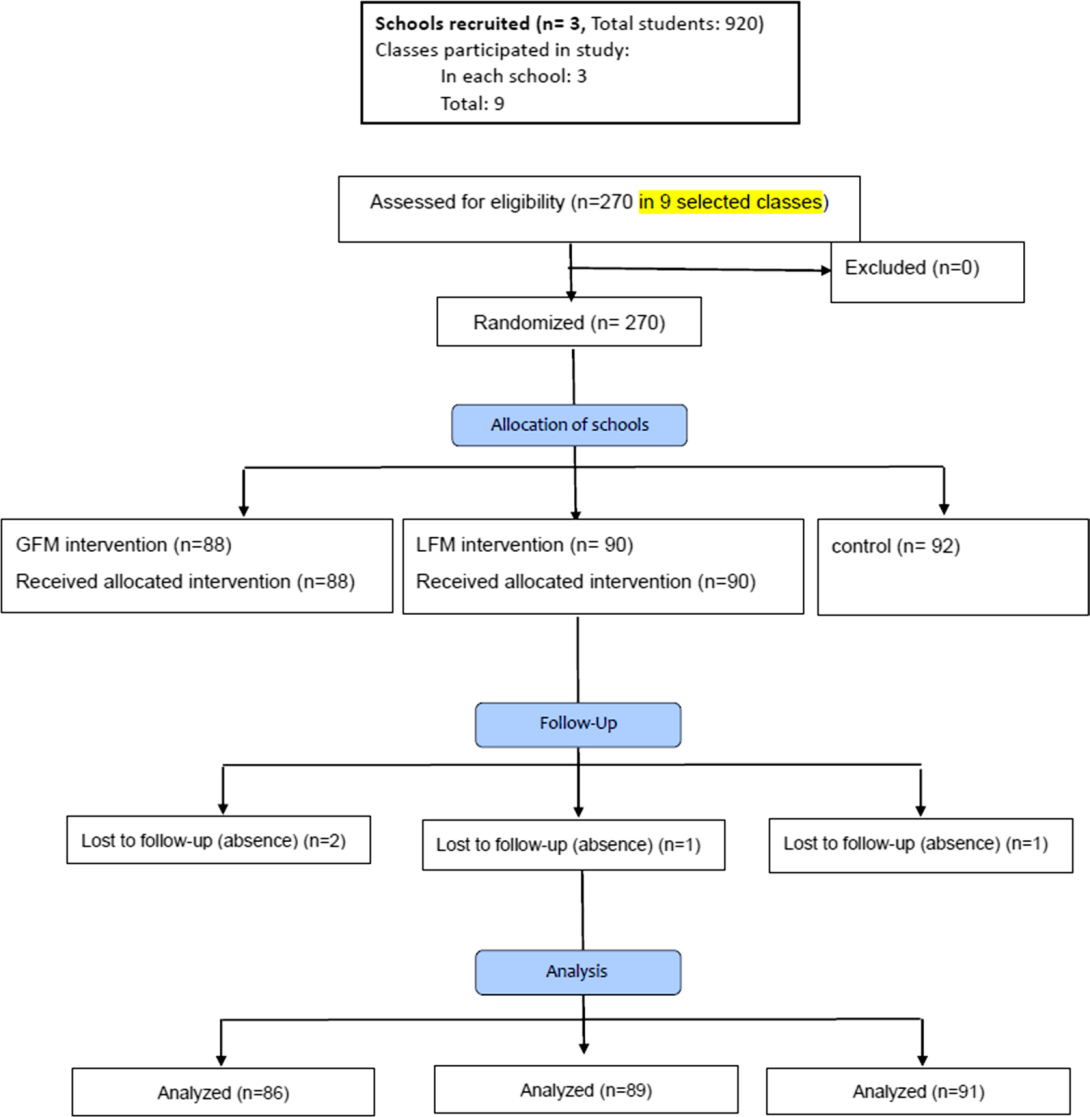
CONSORT flow diagram of participants through the study

This quasi-experimental study with a two-month follow-up was conducted on high-school male students (grades 9–11) in Yazd, Iran, 2015. Considering *α* = 0.05, 1−*β* = 0.80, and a 3–5% attrition rate and using NCSS PASS software, a 250-subject sample size was estimated for the study. At first, three boys’ high schools were selected randomly from an official list of 12 public high schools (with 200–400 students in grades 9–12). In the second step, a class from each grade (9–11) was selected randomly in each school (the 12th grade students were excluded from the study because their school year ended earlier than other grades, which precluded post-testing). All students of the selected classes participated in the study. In order to reduce the risk of data contamination, each of the schools was randomly allocated to one of the three study groups, i.e., gain-framed message, loss-framed message, and control (Fig. 2).

This study was approved and supported by Shahid Sadoughi University of Medical Sciences, Yazd, Iran (Reference Number: 2625). Participation in this study was voluntary, the students received no incentives and they provided informed consent forms for taking part in the research. The only exclusion criterion was lack of willingness to participate in the study.

For data collection, a researcher-designed, self-report, 15-item questionnaire was developed to assess the EPPM cognitive constructs (perceived susceptibility, perceived severity, perceived response efficacy, and perceived self-efficacy) and the intentions about SSBs consumption. Each construct was assessed by three items responded via a five-point Likert scale (from strongly agree to strongly disagree) (Fig. 2). Kim Witte’s 1995 guideline of risk behavior diagnosis scale was used for developing the questionnaire and calculating the participants’ scores [30]. In order to calculate the scores, first the sum of scores of the items of each construct of the EPPM model was calculated. Then, the sum of the scores of perceived susceptibility and perceived severity constructs was calculated for perceived threat and the sum of the scores of perceived response efficacy and perceived self-efficacy was calculated for perceived efficacy. Face and content validity of the questionnaire were approved by an expert panel. In addition, the calculated Cronbach’s alpha (> 0.7) for each construct showed the acceptable internal consistency of the designed tool [31]. Its external consistency was also measured and confirmed by test–retest Pearson’s correlation analysis (*n* = 25, *r* = 0.79, *p* = 0.01). The participants’ daily SSBs consumption was assessed through a single question (during the past month, how many glasses of SSBs did you consume every day?). The questionnaire took approximately 10 min to complete by each student.

After randomization, the baseline data were collected through completing the questionnaires by the participants. Then, each of the two intervention groups received health recommendations by one of the two types of designed pamphlets. In loss-framed pamphlets, the adverse health effects of SSBs, such as obesity, diabetes, dental caries, gastro-esophageal reflux, sleep disorders, hypertension, palpitation, and addiction to SSBs consumption, were emphasized. Gain-framed pamphlets, on the other hand, focused on the benefits of other drinks, such as water, dough (a traditional Iranian drink made by mixing yoghurt with chilled or iced water), tea, coffee, and natural juice. These benefits included supplying minerals and vitamins, helping better food digestion and absorption, lowering serum cholesterol levels, and reducing stress. These pamphlets also contained some practical advice, which helped choose healthier beverages, such as not keeping SSBs at home, using smaller glasses for drinking SSBs, and carrying bottled water on hot days. The students were given half an hour to read the pamphlets in their classes and they could take them home if they wished to. A copy of the pamphlet was also installed in the classroom and was visible to the students for one month. The pamphlets were delivered to students by a MSc in health education. The control group received no pamphlets or recommendations. Two months after the distribution of pamphlets, the participants in the intervention and control groups were asked to complete the questionnaires again.

All statistical analyses were performed using the SPSS 21 software [32]. The normality assumption of the variables was assessed and confirmed by Kolmogorov–Smirnov test (*p* = 0.2). Descriptive analysis, paired t test, one-way ANOVA, and Tukey's post hoc HSD test were used for statistical analyses. Statistical significance was set at *p* < 0.05.

## Findings

This study was conducted on 270 students. The frequency distribution of the participants in each group separated by educational grade is presented in Table 1.

**Table 1:** Distribution frequency of study participants by grade

Group	Grade	*N*	%
Control	9th	33	36.3
	10th	31	34.1
	11th	27	29.7
Gain frame message (GFM)	9th	30	34.9
	10th	28	32.6
	11th	28	32.6
Loss frame message (LFM)	9th	33	37.1
	10th	29	32.6
	11th	27	30.3

In within group comparisons, no significant differences were observed in the mean scores of the study variables in the control group before and after the intervention. However, after the intervention, a statistically significant increase was observed in perceived efficacy and perceived threat in the two intervention groups and a statistically significant decrease was found in their intention to consume SSBs. Despite the decrease in the daily consumption of SSBs in all study groups, the decrease was not statistically significant in any of the three groups (Table 2).

**Table 2:** Comparing Mean Scales of the EPPM constructs before and after the intervention within control, GFM and LFM groups

Group	Variable	Before Mean (SD)	After Mean (SD)	P*
Control	Perceived efficacy	19.66 (5.08)	20.29 (4.98)	.26
	Perceived response efficacy	10.07 (2.69)	10.40 (2.78)	.26
	Perceived Self-efficacy	9.59 (3.36)	9.89 (3.05)	.44
	Perceived threat	18.80 (3.14)	18.95 (3.72)	.71
	Perceived susceptibility	8.43 (2.55)	8.17 (2.16)	.35
	Perceived severity	10.19 (1.94)	10.76 (2.73)	.93
	Intention	9.25 (3.80)	9.63 (2.51)	.37
	Daily SSBs consumption (glasses)	2.95 (2.54)	2.43 (1.87)	.13
Gain frame message	Perceived efficacy	19.62 (4.51)	22.43 (4.08)	< .001
	Perceived response efficacy	9.79 (2.56)	11.31 (2.48)	< .001
	Perceived Self-efficacy	9.80 (2.65)	11.12 (2.39)	< .001
	Perceived threat	18.36 (3.06)	19.32 (3.47)	.023
	Perceived Susceptibility	7.72 (2.88)	7.57 (2.35)	.66
	Perceived severity	10.64 (1.22)	11.74 (2.57)	.001
	Intention	9.09 (3.56)	9.00 (3.69)	.83
	Daily SSBs consumption (glasses)	2.31 (2.06)	2.12 (2.11)	.54
Loss frame message	Perceived efficacy	20.24 (4.54)	22.57 (4.78)	< .001
	Perceived response efficacy	10.45 (2.43)	11.60 (2.47)	< .001
	Perceived Self-efficacy	9.84 (3.37)	10.95 (3.26)	.021
	Perceived threat	18.01 (2.79)	21.26 (3.67)	< .001
	Perceived Susceptibility	7.85 (2.28)	8.34 (2.12)	.07
	Perceived severity	10.19 (1.52)	12.86 (2.69)	< .001
	Intention	8.90 (3.19)	8.05 (3.04)	.04
	Daily SSBs consumption (glasses)	3.00 (3.01)	2.38 (1.73)	.16

*Paired *t* test

In between-group comparisons, no significant differences were observed among the three study groups regarding the mean scores of cognitive constructs (perceived response efficacy, self-efficacy, perceived susceptibility, and perceived severity), intention to consume SSBs, and daily consumption of SSBs before the intervention (*p* > 0.05). After the intervention, however, the gain-framed messaging group had higher perceived self-efficacy compared to the control group and a lower perceived severity compared to the loss-framed messaging group. Moreover, the constructs of perceived response efficacy and perceived severity increased significantly and the intention to consume SSBs reduced significantly in the loss-framed messaging group compared to the control group. The mean score of perceived threat was also significantly higher in the loss-framed messaging group in comparison to the control and gain-framed messaging groups (*p* < 0.001). However, no significant difference was detected between the gain-framed messaging and control groups with respect to perceived threat. Furthermore, the mean score of perceived efficacy was significantly higher in the two intervention groups in comparison to the control group, but no significant difference was observed between the gain-framed and loss-framed messaging groups in this respect (*p* < 0.001) (Table 3).

**Table 3:** Between groups comparison of study variables’ mean scales, after the intervention

Variable	One-way ANOVA		Post hoc (Tukey HSD) Pairwise intergroup comparison		
	*F*	*P*	Control-GFM	Control-LFM	GFM-LFM
			*F*	*F*	*F*
Perceived efficacy	5.65	.004	−2.13*	−2.05*	0.08
Perceived response efficacy	4.65	.01	−0.91	−1.16*	−0.25
Perceived Self-efficacy	3.82	.02	−1.22*	−0.87	0.35
Perceived threat	9.76	< .001	−0.37	−2.34*	−1.96*
Perceived Susceptibility	2.60	.076	0.56	−0.22	−0.78
Perceived severity	12.43	< .001	−0.93	−2.05*	−1.12*
Intention	3.11	.046	0.63	1.34*	0.71
Daily SSBs consumption(glasses)	7.36	.48	0.34	0.05	−0.29

*SSBs* sugar-sweetened beverages, *GFM*  gain frame message, *LFM* loss frame message

*p < .001

## Discussion

This study aimed to investigate and compare the effects of two types of health messages (gain framed and loss framed) on the intention to consume SSBs and their consumption among high school boy students in Yazd, Iran. Although perceived self-efficacy and perceived response efficacy were increased in both of the intervention schools, mean score of perceived severity was increased and intention to consume SSBs was decreased significantly in the school receiving message with the threatening theme. On the other hand, both perceived threat and perceived efficacy mean scores were increased in the two intervention groups, while perceived susceptibility did not change significantly. It should be noted that although the changes in some cases such as perceived threat in the group receiving the gain frame message and self-efficacy in the group receiving the loss frame message were statistically significant, they may not be clinically significant.

The theoretical foundations of the EPPM suggest that high perceived efficacy (self-efficacy + response efficacy) associated with high perceived threat (perceived Susceptibility + perceived severity) lead to danger control responses and acceptance of proposed recommendations in health messages. Evidence has indicated that the effectiveness of loss-framed and gain-framed messages in health-related behavior change depended on the nature of the behavior and the risk ratio of its complications. For instance, gain-framed messages were more effective in persuading the prevention of low-risk behaviors, while loss-framed ones were more appropriate for high-risk behaviors. Thus, people were more persuaded by the information they received about the advantages of eating healthy food compared to the messages they received about the complications of eating unhealthy food [25]. According to the prospect theory also, positive messages were more persuasive than negative ones in promoting preventive behaviors (such as doing exercise and nutrition) [35]. However, various studies regarding the effect of gain- and loss-framed messages have not reached a single conclusion. For example, Okeefe et al. [26] believed that there was no evidence that positive or negative messages would have a persuasive role in influencing eating behaviors as well as in reducing obesity. Godinho et al. [27] also conducted a study on students and concluded that although gain-framed messages were of higher quality from the participants’ viewpoints, they were not significantly different from loss-framed messages regarding their effects on the intention to consume fruits and vegetables. They pointed to other important factors, such as audiences’ motivational orientation, in the effectiveness of messages, as well. On the other hand, Moscato et al. [28] investigated on Greek students and reported that fear appeal messages might be a useful way to control the consumption of alcoholic drinks. Pakpour et al. [29] also performed a study on Iranian adolescents and disclosed that loss-framed messages were more effective than gain-framed messages in addressing oral health behaviors. The poor effect of gain-framed messages on the prevention of low-risk behaviors such as diet has been demonstrated, as well [25, 33]. In the present study, the group receiving loss-framed messages showed a significant decrease in the intention to consume SSBs, while such a change was not observed in the group receiving gain-framed messages. In contrast, Robert et al. [22] claimed that information with an emphasis on perceived severity was the least persuasive component of loss-framed messages and could lead to defensive reactions, such as risk denial, biased information processing, and less attention to health promotion messages. The findings of the research by Napper et al. [34] also showed that efficacy and threat × efficacy interaction were the significant predictors of the motivation to consume more fruits and vegetables. In the same vein, Gallagher et al. [33] found in a meta-analysis that encouraging messages were more effective in preventive behaviors. Similarly, Zahid and Reicks [35] indicated in a study that the parents who received encouraging messages were more motivated to control SSBs consumption in their children compared to those who had received threatening messages.

Over all, the present study results revealed that each of the two types of the presented messages influenced the cognitive constructs under investigation regarding the consumption of SSBs. In addition, the threatening messages were associated with a decrease in the intention to consume SSBs. Nonetheless, the daily consumption of SSBs was not affected by any of the two message types.

The main strengths of the current study were the use of a theory-based approach in designing the educational intervention and its three-arm design. However, the study had some limitations. One of the study limitations was that the participants were limited to urban boy students in grades 9–11. Therefore, the results might not be generalized to rural and girl students or those in other educational grades. Another limitation of the study was that the data were collected as self-report and no method was considered for observing and recording the students’ behaviors. Using only one education method, and assigning only one school to each treatment arm was yet another limitations of the present study. It is worth noting that, while quasi-experimental designs are often applied in public health because of ethical or feasibility considerations, one of the main limitations of these studies is their ability to attribute observed changes to intervention. Finally, face validity of the study questionnaire was assessed only through the panel of experts and the participants were not included in this process.

## Conclusion

The results of the present study indicated that increasing self-efficacy and perceived response efficacy alone could not decrease the intention to consume SSBs. The results supported the utilization of health messages to increase the level of perceived threat, especially perceived severity, of the complications of high-risk behaviors. Thus, a combination of loss-framed and gain-framed messages could lead to nutritional behavior change regarding the consumption of SSBs more effectively.

## Data Availability

Please contact author for data requests.

## Abbreviations

EPPM: Extended parallel process model
GFM: Gain frame messages
LFM: Loss frame messages
NCD: Non-communicable diseases
SSB: Sugar-sweetened beverages
